# MEK1 is required for the development of NRAS-driven leukemia

**DOI:** 10.18632/oncotarget.12555

**Published:** 2016-10-10

**Authors:** Joanna D. Nowacka, Christian Baumgartner, Cristiana Pelorosso, Mareike Roth, Johannes Zuber, Manuela Baccarini

**Affiliations:** ^1^ Department of Microbiology and Immunobiology, Max F. Perutz Laboratories, University of Vienna, Vienna, Austria; ^2^ Research Institute of Molecular Pathology, Vienna, Austria; ^3^ Pediatric Neurology, Neurogenetics and Neurobiology Unit and Laboratories, A. Meyer Children's Hospital–University of Florence, Florence, Italy

**Keywords:** MEK1, leukemia, NRAS, MYC

## Abstract

The dual-specificity kinases MEK1 and MEK2 act downstream of RAS/RAF to induce ERK activation, which is generally considered protumorigenic. Activating MEK mutations have not been discovered in leukemia, in which pathway activation is caused by mutations in upstream components such as RAS or Flt3. The anti-leukemic potential of MEK inhibitors is being tested in clinical trials; however, downregulation of MEK1 promotes *Eμ-Myc*-driven lymphomagenesis and MEK1 ablation induces myeloproliferative disease in mice, raising the concern that MEK inhibitors may be inefficient or counterproductive in this context. We investigated the role of MEK1 in the proliferation of human leukemic cell lines and in retroviral models of leukemia. Our data show that MEK1 suppression via RNA interference and genomic engineering does not affect the proliferation of human leukemic cell lines in culture; similarly, MEK1 ablation does not impact the development of MYC-driven leukemia *in vivo*. In contrast, MEK1 ablation significantly reduces tumorigenesis driven by *Nras* alone or in combination with *Myc*. Thus, while MEK1 restricts proliferation and tumorigenesis in some cellular and genetic contexts, it cannot be considered a tumor suppressor in the context of leukemogenesis. On the contrary, its role in NRAS-driven leukemogenesis advocates the use of MEK inhibitors, particularly in combination with PI3K/AKT inhibitors, in hematopoietic malignancies involving RAS activation.

## INTRODUCTION

Leukemias are genetically and biologically heterogeneous hematopoietic malignancies characterized by the expansion of transformed cells which interferes with the proper differentiation and maturation of all blood lineages. Leukemia can be categorized based on the lineage origin of malignant cells and their maturation status, forming four major groups that account for 70% of all human leukemia: acute myeloid leukemia (AML), acute lymphoblastic leukemia (ALL), chronic myeloid leukemia (CML), and chronic lymphocytic leukemia (CLL) [1]. Neoplastic transformation is initiated by activating mutations of proto-oncogenes or loss-of function mutations in tumor suppressor genes occurring in the hematopoietic stem cells (HSCs) or multipotent progenitor cells (MPPs) [2].

The RAF/MEK/ERK pathway is a central regulator of fundamental cellular processes including cell proliferation, survival, differentiation, apoptosis, motility and metabolism. It is activated by the small GTPase RAS downstream of growth factor and cytokine receptors and plays a key role in the development. Deregulation of the pathway has been shown to lead to various developmental disorders, as well as carcinogenesis [3, 4]. In particular, the RAS/RAF/MEK/ERK pathway contributes to a number of myeloproliferative disorders [5, 6], to myelodysplastic syndrome (MDS) [7], and to hematopoietic malignancies [8]. Mutational activation of the ERK cascade may occur at multiple levels - at the receptor (for example FLT3, KIT, PDGFP), at GTPases (RAS) or at the kinase cascade itself (RAF); additionally, the ERK pathway can be activated by fusion proteins resulting from chromosomal translocations such as BCR-ABL. Consistent with this, ERK is activated in 83% of AML [9] and 35% of ALL [10]. However, while activating mutations in the upstream components of the pathway are quite frequent in cancer, mutations of MEK1/2 are very sporadic. To date, only a few mutations that increase the activity of MEK1 have been described in ovarian cancer, lung cancer, melanoma and colon cancer (recently reviewed in [11]). Several studies have shown that overexpression of active MEK1 is transforming [8], but gain-of-function mutations in MEK or ERK have not been detected in leukemia samples. MEK inhibitors and other pathway inhibitors are in clinical trials and have moderate efficacy as single agents in AML and ALL, particularly in leukemias harboring RAS pathway mutations; synergy has been shown with compounds inhibiting other signaling pathways including the PI3K/AKT pathway [12, 13].

MEK1 and MEK2 are dual-specificity kinases that share a common mechanism of activation by RAF and a common target, ERK1/2. Due to their high structural similarity, common mechanisms of activation, and common substrates, MEK1 and MEK2 have traditionally been considered functionally redundant. Conditional ablation experiments, however, have revealed that MEK1 plays an essential role in the regulation of strength and duration of MEK2/ERK [14] and AKT signals [15]. Mice carrying an epiblast-restricted deletion of the *Mek1* gene are born at Mendelian ratios without any gross abnormalities, although higher ERK1/2 activation levels can be observed in the embryos as well as in the epidermis and brain of young mice [14]. However, MEK1 KO mice show decreased survival rate and develop myeloproliferative and lupus-like autoimmune disorders associated with AKT activation in hematopoietic cells and tissues [15]. In addition, data from an *in vivo* RNA interference screen for tumor suppressor genes in a mouse Eμ-MYC-driven lymphoma model have revealed that downregulation of MEK1 accelerates the development of this disease. Although the exact mechanism has not been delineated, together these results suggest that MEK1 may suppress proliferation and even tumorigenesis in the context of the hematopoietic system, raising concerns about the safety of treatments inhibiting MEK in leukemia. Against this backdrop, we set out to investigate the impact of MEK1 ablation on the initiation and promotion of leukemogenesis.

## RESULTS & DISCUSSION

### MEK1 down-regulation confers no selective advantage to human leukemia cell lines

**Figure 1:**
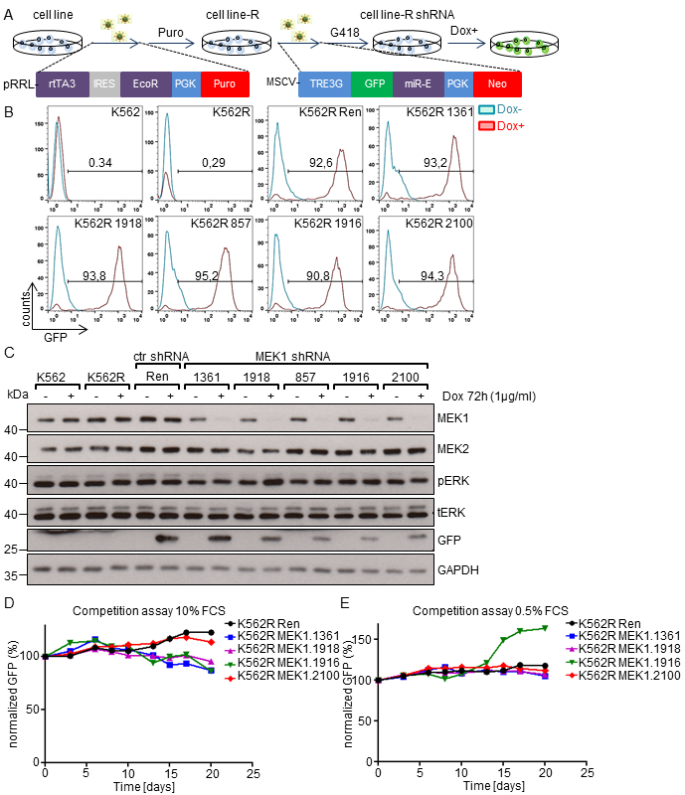
MEK1 knock-down does not confer a selective advantage to human K562 leukemia cell line **A.** Generation of MEK1 knock-down cell lines. K562 cells were transduced with lentiviral particles expressing a Tet responsive transactivator (rtTA3) and ecotropic receptor. After puromycin selection the transduced cells (K562R) were infected with retroviral particles carrying a GFP reporter and shRNAs against the *MEK1* gene embedded in a microRNA stem (miR-E)(shRNA #1361, #1916, #857, #1918, #2100). The GFP-miR-E cassette is under the control of an inducible tetracyclin responsive promoter and its expression is activated by doxycyline (Dox) treatment. **B.** FACS analysis of batch cultures showing strong expression of the GFP-mir-E cassette. The numbers indicate the percentage of GFP^+^ cells. **C.** Efficient knock-down of MEK1 induced by Dox treatment in established cell lines. GAPDH is shown as a loading control. tERK, total ERK; pERK, phosphorylated ERK. The Western blot is representative of 3 independent experiments. **D.** and **E.** Effect of MEK1 KD on cell proliferation in competition assays. Dox pre-treated K562R Ren ctr or MEK1 KD cells expressing shRNAs and GFP reporter were mixed in 1:1 ratio with K562R cells (no GFP). Cells were cultured in the presence of 10% (D) or 0.5% FCS (E) and passaged every 2-3 days, depending on confluency. After each passage, an aliquot of the co-culture was evaluated for the % of GFP^+^ cells and normalized to the initial GFP^+^ cells content on day 0.

We employed RNAi technology to knock-down (KD) MEK1 in human leukemia cell lines and investigate its role in their proliferation. We used a previously described short-hairpin RNA (shRNA) Tet-ON system (Figure 1A), in which leukemic cells are first transduced with lentiviral pseudoparticles carrying the reverse tetracycline-controlled transactivator (rtTA3) and an ecotropic receptor [17]. The established cell lines (denoted as R) were further infected with retroviral vectors encoding Tet-inducible MEK1 shRNAs embedded in the optimized microRNA stem (miR-E) located in the 3'UTR of GFP gene [18].

We first tested the effect of MEK1 suppression in K562 cell lines. The human erythromyeloblastoid leukemia cell line K562 expresses the BCR-ABL fusion protein, known to initiate the ERK pathway by direct activation of RAS proteins [19, 20]. Flow cytometry (FACS) analysis of the established batch-cultures revealed that more than 90% of cells expressed the GFP-miR-E cassette upon doxycycline treatment (Figure 1B). Tet-induced GFP expression and efficient downregulation of MEK1 by all shRNAs were verified by immunoblotting (Figure 1C). ERK expression and phosphorylation were not affected by MEK1 knock-down (Figure 1C).

We next investigated the impact of MEK1 down-regulation on K562 cells proliferation in a competition assay. K562 MEK1 KD cell lines were pre-treated with doxycycline, mixed with the parental K562R cells and co-cultured in medium supplemented with 10% or 0.5% FCS (Figure 1D, 1E). Although the percentage of GFP-miR-E expressing cells in all four MEK1 KD cell lines tested showed some variation, MEK1 suppression had no major effect on proliferation and survival compared to the control Ren shRNA cell line. The only exception was one of the four MEK1 shRNA (MEK1.1916) clones, which proliferated faster than all others in the low serum (0.5%). This effect was not observed in the remaining 3 cell lines, and therefore likely resulted from shRNA specific off-target effects or clonal variation.

We applied the same strategy to knock-down MEK1 in the THP-1 human monocytic leukemia cell line, harboring the NRAS^G12D^ mutation and a *MLL-AF9* fusion [21, 22]. In these cells, NRAS^G12D^ is predicted to constitutively activate MEK/ERK [23]. In addition, MLL-AF9 has been shown to directly bind to the promoter of erythropoietin-producing hepatoma-amplified sequence (*epha7*) receptor tyrosine kinases, leading to its upregulation and subsequent MEK-dependent ERK phosphorylation [24].

**Figure 2:**
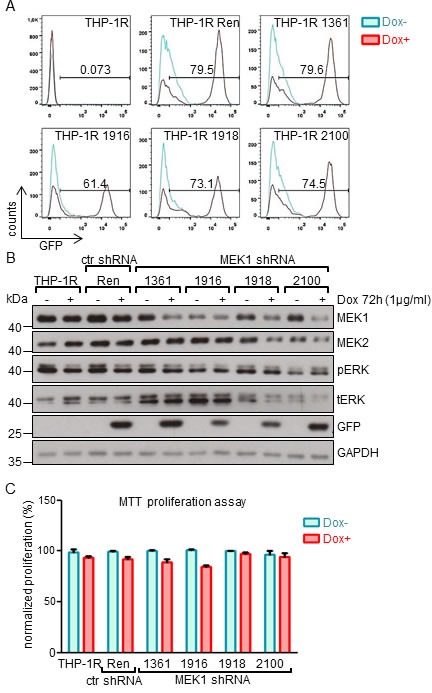
MEK1 knock-down has no impact on the proliferation of human THP-1 leukemia cell line expressing NRAS^G12D^ and MLL-AF9 oncogenes **A.** MEK1 KD cells were generated as described in the legend to Figure 1. FACS analysis of batch cultures showing strong expression of the GFP-mir-E cassette. The numbers indicate the percentage of GFP^+^ cells. **B.** MEK1 knock-down efficiency in established cell lines after 72h of Dox-induced expression of shRNA. GAPDH is shown as a loading control. tERK, total ERK; pERK, phosphorylated ERK. The Western blot is representative of 3 independent experiments. **C.** MTT proliferation assay shows no significant difference between the proliferation of WT and KD cells after 7 days in culture.

The results were similar to those obtained using the K562 cell line. We observed Tet-regulated expression of GFP in batch cultures (more than 75% of the cells positive for the expression of GFP-miR-E cassette in shRNA 1361, 1918 and 2100 lines; 61% in the 1916 lines; Figure 2A) and efficient down-regulation of MEK1 protein levels (Figure 2B). Clonal variation could be observed in both ERK expression (upregulated in shRNA 1361 and 1916 lines) and phosphorylation (slight upregulation in shRNA 1918 and 2100 lines (Figure 2B). However, MEK1 down-regulation did not affect the proliferation of THP-1 cells (Figure 2C).

RNAi technology enables the generation of hypomorphic expression states, which are distinct from a complete KO. We therefore applied CRISPR-Cas9 technology [25] to knock-out MEK1 in HL-60 promyelocytic leukemia cells, which in addition to the NRAS^Q61L^ mutation harbors an amplification of MYC (CMYC) [26], whose enforced expression was shown to cooperate with MEK1 silencing in B cell leukemogenesis [16].

**Figure 3:**
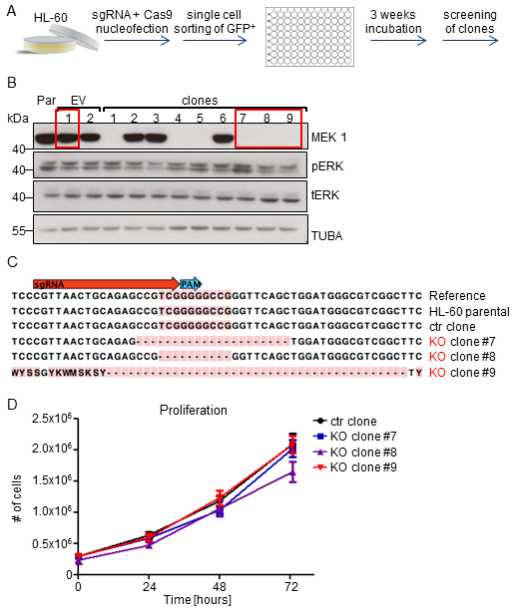
MEK1 knock-out has no impact on the proliferation of the human HL-60 leukemia cell line carrying MYC amplification and expressing NRAS^Q61L^oncogenes **A.** Schematic of CRISPR-Cas9 mediated MEK1 KO: HL-60 human leukemia cells were transiently nucleofected with a vector carrying Cas9-GFP and a single guide RNA targeting exon 1 of ***MEK1*** gene. After 3 weeks of incubation, single cell clones were screened for successful MEK1 ablation. **B.** Two thirds of the clones screened showed successful MEK1 KO. The Western blot is a representative of 3 independent experiments. Tubulin A is shown as a loading control. tERK, total ERK; pERK, phosphorylated ERK. Par, HL-60 parental cell line; EV, HL-60 cells transfected with Cas9 vector without RNA guides. The red boxes show the clones chosen for further experiments. **C.** Sequencing of selected clones revealed efficient target site cleavage. **D.** Proliferation assay shows no significant difference between proliferation of control (ctr) and KO clones.

HL-60 cells nucleofected with a single guide RNA (sgRNA) against *MEK1* exon1 and a Cas9-GFP fusion protein were FACS-sorted and grown as single cell clones (Figure 3A). Immunoblot analysis showed successful MEK1 knock-out in 2 out of 3 screened clones (Figure 3B). Indel formation in the sgRNA target site was confirmed by sequencing (Figure 3C). ERK expression was slightly increased in clones 8 and 9, while clone 7 showed increased ERK phosphorylation. However, we did not observe any differences in the proliferation rate of the control cells (wild type (WT) cells transfected with Cas9 only) and knock-out (KO) clones (Figure 3D).

Together, the data in Figures 1-3 indicate that MEK1 knock-down or knockout does not correlate with increased ERK phosphorylation in three human leukemia cells lines; a potential explanation of this fact is that output and feedback might both be reduced in MEK1-deficient cells, resulting in (nearly) equal levels of pERK. In addition, neither knock-down nor knock-out of MEK1 influenced the proliferation of human leukemia cell lines harboring oncogenic changes leading to the activation of the ERK pathway. All these cell lines, however, also contain loss of function mutations of the *TP53* tumor suppressor gene [27]. It is possible that in the context of so many genomic alterations, the impact of MEK1 down-regulation is marginal. Alternatively, MEK1 might not influence the progression of the established disease, but rather play a role in its initiation.

### MEK1 ablation cooperates with MYC oncogene in semisolid culture

**Figure 4:**
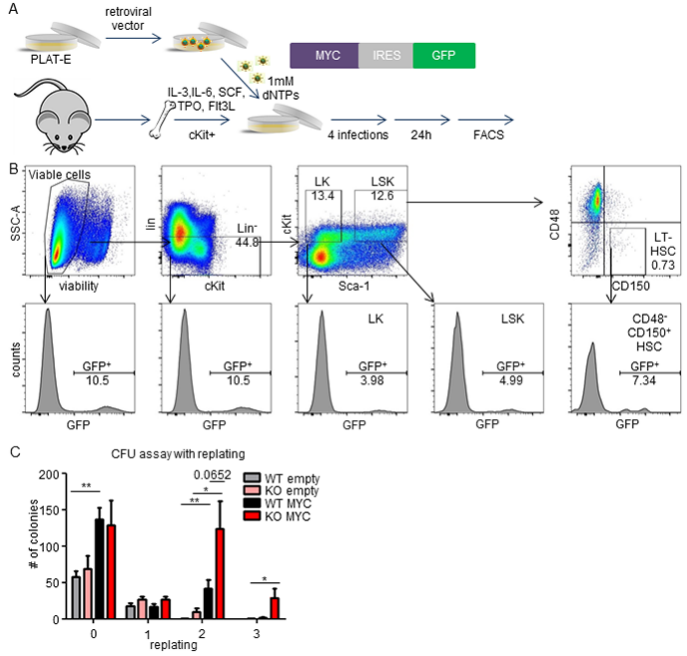
Cooperation of MEK1 ablation with MYC overexpression in semisolid culture **A.** Schematic of the retroviral transduction protocol. Retroviruses carrying an expression cassette containing *Myc* and a GFP reporter were produced in Platinum-E packaging cells and used for infection of c-Kit-enriched BM cells. **B.** Successful transduction of hematopoietic stem cells. Top panel: gating strategy; from left to right, viable cells, Lin- cells, committed progenitors (LK), multipotent progenitors and stem cells (LSK). The bottom panels show the % of GFP^+^ cells in the boxed areas of the top panel. **C.** Colony forming unit (CFU) assay with MYC transduced *Mek1* (WT) or *Mek1; Sox2Cre*(KO) cells indicating cooperation of MEK1 ablation and MYC in a replating assay measuring self-renewing potential.

To investigate the role of MEK1 in the initiation of leukemia, we first examined the cooperation of MEK1 ablation with MYC in culture. To this end, we developed an optimized protocol for the transduction of cKit^+^ bone marrow hematopoietic stem and progenitor cells (BM HSPCs), which were shown to be the cells of origin in various leukemia models [28-30]. BM HSPCs were cultured in a cytokine cocktail containing IL-3, IL-6, SCF, TPO and Flt-3 ligand [31]. After 18 hrs in culture, cells were infected with retroviral pseudoparticles encoding an expression cassette harboring the mouse *Myc* gene and a GFP reporter in medium supplemented with 1mM dNTPs to improve retroviral reverse transcription, inefficient in quiescent BM cells due to their low dNTP levels [32] (Figure 4A). FACS analysis showed successful transduction of both committed progenitor cells (LK; lin^−^, cKit^+^, Sca1^−^) and multipotent progenitor and stem cells (LSK; lin^−^, cKit^+^, Sca-1^+^; Figure 4B). Further discrimination of the LSK population using CD48 and CD150 markers revealed enhanced transduction of the LSK, CD48^−^, CD150^+^ subpopulations, enriched for hematopoietic stem cells.

Next, we used methylcellulose colony assays to assess the impact of MEK1 ablation on the repopulation capacity of *Myc*-transduced BM HSPCs. Overexpression of MYC increased the number of colony forming cells (CFCs) approx. 2.5 fold in both MEK1 WT and KO cells (Figure 4C). After the first replating, the number of CFCs decreased drastically in all tested conditions. However, *Myc* transduced MEK1 KO cells performed significantly better than their WT counterpart in the second replating, and were the only population able to proliferate in the next round. These data are in line with the previously published observation that MYC represses the self-renewing capacity of HSCs and does not stimulate the colony-forming activity of myeloid progenitors [33, 34]. However, the increased numbers of CFCs produced by MEK1 KO MYC cells in the second and third replating suggested that MEK1 ablation cooperates with MYC and enhances the repopulating potential of *Myc* transduced cells.

### MEK1 ablation does not impact the survival or the disease spectrum in a MYC-driven leukemia model

**Figure 5:**
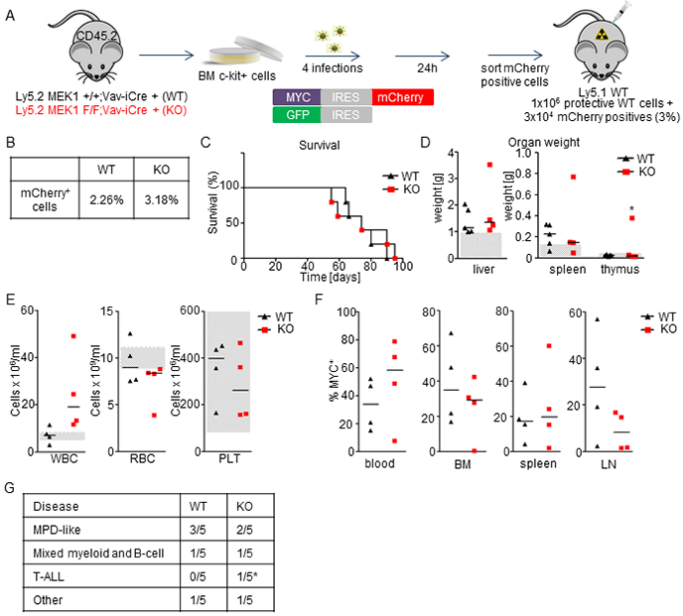
MEK1 ablation does not significantly impact survival or disease spectrum in a MYC-driven leukemia model **A.** Schematic of experimental procedure. c-Kit^+^-enriched BM cells were transduced with retroviral particles coding for *Myc* and mCherry. 24h after the last infection, MYC^+^ cells were sorted, mixed with protective BM and injected in lethally irradiated WT mice (3x10^4^ WT or KO MYC^+^ cells in 1x10^6^ protective WT BM per mouse). **B.** Initial transduction efficiency. **C**. Kaplan-Meier plot depicting the survival of recipients transplanted with WT or MEK1 KO MYC^+^ cells. **D**. Moderate hepatomegaly and splenomegaly in mice transplanted with both WT and KO MYC^+^ cells. Grey boxes indicate the physiological weight range. **E**. Mice transplanted with KO MYC^+^ cells show a tendency towards increased white blood cells (WBC) counts and more profound anemia (RBC) compared to the mice transplanted with WT MYC^+^ cells. Platelets (PLT) counts are similar and in the normal range. Grey boxes indicate the physiological range. **F**. Moribund animals transplanted with either WT or KO MYC^+^ cells show expansion of MYC^+^ cells in blood, BM, spleen, and lymph nodes (LN). **G**. A broad spectrum of diseases develop in mice transplanted with both WT and KO MYC^+^ cells. The asterisk in **D** and **J** indicates one mouse with T-ALL.

We next tested the impact of MEK1 ablation on the initiation and progression of MYC-driven leukemia. Lethally irradiated mice were transplanted with MEK1 WT or KO BM cells transduced with *Myc* (3% in protective WT BM; Figure 5A, 5B). All mice injected with WT MYC^+^ cells succumbed to the disease within 3 months (Figure 5C) and developed mild to medium hepatomegaly and splenomegaly (Figure 5D). The expansion of MYC^+^ cells was evident in blood (ranging from 10-60%), BM (up to 70%), spleen (up to 40%) and lymph nodes (5-60%) (Figure 5F). FACS analysis revealed that leukemic cells expressed a range of lineage markers that varied significantly between individual mice but belonged mostly to myeloid and B cell lineage (Figure 1G and Supplementary Figure 1). The ablation of MEK1 did not accelerate leukemogenesis (median survival for both groups = 74 days; Figure 5C), nor did it lead to major changes in the disease spectrum (Figure 5G and Supplementary Figure 1). Compared to the WT MYC^+^ cohort, the animals transplanted with KO MYC^+^ cells showed a tendency towards increased white blood cells counts and more profound anemia (Figure 5E); one out of five mice transplanted with KO MYC^+^ cells presented with an enlarged thymus with accumulation of transformed, undifferentiated T cells (Figure 5D and data not shown). In conclusion, mice transplanted with either MEK1 WT or KO MYC overexpressing cells developed a broad spectrum of diseases (MPD-like, mixed myeloid and B cells, T-ALL).

Multiple studies have demonstrated the leukemogenic potential of enforced overexpression of MYC using retroviral vectors; however, the results of these studies vary dramatically depending on the cell type used in the infection. MYC overexpression in 5'FU treated BM cells was sufficient to promote the development of rapid AML [35], while in another study using lineage-depleted BM cells it failed to do so [33]. On the other hand, fetal liver cells transduced with *Myc* retroviruses produced low-frequency, long-latency lymphomas [36]. In our setting, overexpression of MYC led to a mixed disease spectrum, probably resulting from leukemogenic transformation of cells in various differentiation stages; MEK1 ablation, however, did not have any significant impact on either survival or disease spectrum.

This finding was unexpected in view of the enhanced repopulating capacity of *Myc* transduced, MEK1 KO BM cells in the CFU assay, as well as in view of the effect of MEK1 downregulation in the Eμ-MYC pre-B lymphoma model [16]. The first discrepancy could be the result of the different experimental conditions (*in vitro* culture vs. exposure to the BM niche). The obvious explanations for the discrepancy between our experiment and the results obtained in the Eμ-MYC pre-B lymphoma model are the difference in the onset and type of the disease; in the lymphoma model, MYC was expressed exclusively in pre-B progenitor cells, while we targeted all lineages causing the development of myeloid and mixed myeloid/B cell malignancies. Additionally, in the system we used the onset of the disease is rapid (2-3 months); in the pre-B lymphoma model, the mice remained disease-free for the period of more than 180 days and MEK1 down-regulation accelerated the onset of the disease to 2-3 months [16]. Finally, in our experiments MEK1 was completely ablated, as opposed to incomplete downregulation by RNAi.

Together, our and previous studies [16] demonstrate that while MEK1 downregulation has protumorigenic effects in certain contexts, MEK1 does not act as a generally relevant tumor suppressor gene in MYC-driven hematopoietic cancers of different origin.

### MEK1 ablation prolongs the survival of mice in leukemia models driven by NRAS^G12D^ and NRAS^G12D^/MYC

We next investigated whether MEK1 ablation would have an impact on the development of leukemia driven by its upstream activator NRAS. Activating NRAS mutations have been observed in 8-11% of AML [37], 20% of juvenile myelomonocytic leukemia (JMML) [38] and 8% of T lymphomas and T leukemias [39], making NRAS the most frequently mutated RAS isoform in both lymphoid and myeloid malignancies.

**Figure 6:**
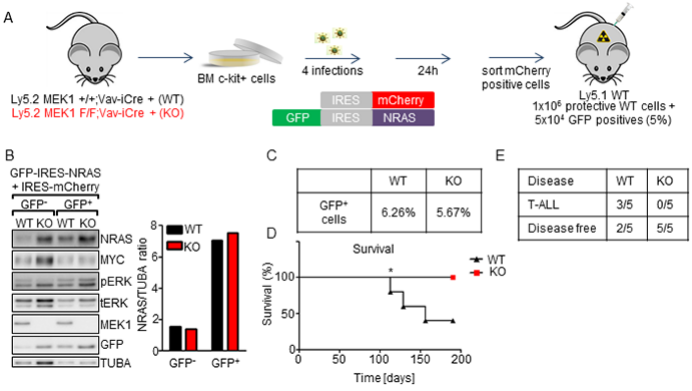
MEK1 ablation prolongs the survival of mice in a NRAS^G12D^-driven leukemia model **A.** Schematic of experimental procedure: c-Kit^+^-enriched BM cells were transduced with retroviral particles coding for the *Nras* oncogene and GFP. 24h after the last infection GFP^+^ cells were sorted, mixed with protective BM and injected in lethally irradiated WT mice (5x10^4^ WT or KO NRAS^G12D+^ cells in 1x10^6^ protective WT BM per mouse). **B.** Efficient, equal upregulation of NRAS in transduced cells. Transduced cells were sorted for GFP^-^ or GFP^+^ cells and analyzed by immunoblotting. The plot represents a densitometric quantification of the immunoblots performed using ImageLab software. The data are expressed as relative band intensity adjusted to TUBA, which serves as a loading control. **C.** Initial transduction efficiency. **D.** Kaplan- Meier plot depicting the survival of recipients transplanted with WT or MEK1 KO NRAS^G12D^ cells. The data were analyzed using the log-rank test. χ2 = 3.862, *p* = 0.0494. **E**. 3 out of 5 WT mice developed T-ALL. The other WT and all KO mice remained disease free.

Lethally irradiated mice were transplanted with MEK1 WT or KO cells transduced with *Nras^G12D^* (5% in protective BM WT (Figure 6A, 6C); we increased the percentage of transduced cells because NRAS^G12D^ was shown to have low leukemogenic potential [40]). Immunoblot analysis showed approximately 4.5-fold NRAS overexpression in GFP^+^ WT and KO cells. Overexpression was accompanied by ERK phosphorylation, slightly higher in the KO (Figure 6B). Three out of five recipients of WT HSPC transduced with *Nras*^G12D^ developed late-onset thymic lymphoma, consistent with previous reports showing that enforced expression of NRAS^G12D^ results in late-onset T-ALL in bone marrow transplantation assays [41, 42] (Figure 6D, 6E). In contrast, all mice transplanted with NRAS^G12D^ KO BM cells remained disease-free throughout the observation period of 200 days (Figure 6D, 6E). Thus, loss of MEK1 prevents NRAS^G12D^-driven leukemogenesis.

**Figure 7:**
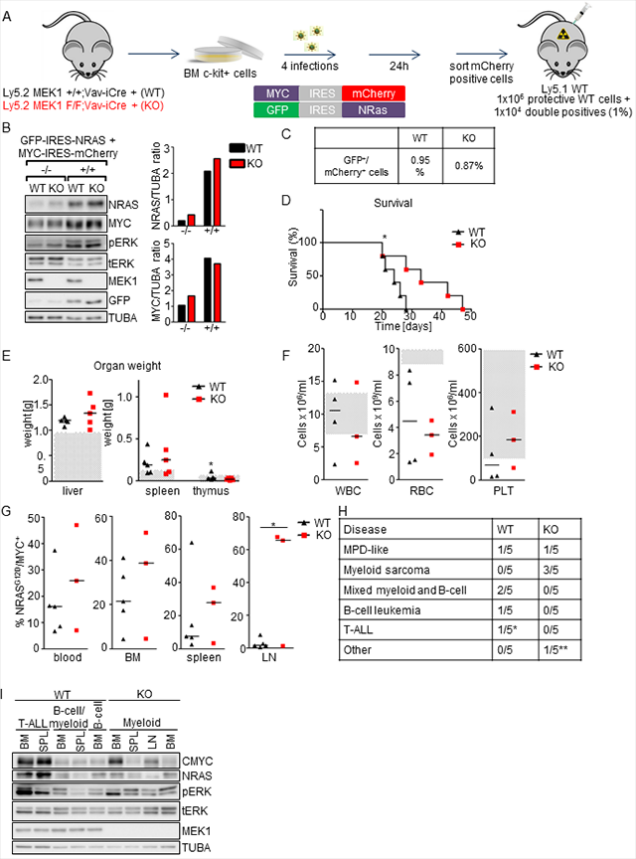
MEK1 ablation prolongs the survival of mice in NRAS^G12D^/MYC-driven leukemia model and shifts the spectrum of the disease towards myeloid malignancies **A.** Schematic of experimental procedure. c-Kit^+^-enriched BM cells were transduced with two retroviral particles coding for the *Nras* and *Myc* oncogenes. 24h after the last infection GFP/mCherry^+^ cells were sorted, mixed with protective WT BM and injected in lethally irradiated WT mice (1x10^4^ WT or KO NRAS^G12D^/MYC^+^ cells in 1x10^6^ WT protective BM per mouse). **B.** Efficient, similar upregulation of NRAS^G12D^ and CMYC in transduced cells. Transduced cells were sorted for GFP^−^/mCherry^-^ (-/-) or GFP^+^/mCherry^+^ (+/+) cells and analyze by immunoblotting. The plots represent a densitometric quantification of the immunoblots performed using ImageLab. The data are expressed as relative band intensity adjusted to TUBA, which serve as a loading control. **C.** Initial transduction frequency. **D.** Kaplan-Meier plot depicting the survival of recipients transplanted with WT or MEK1 KO NRAS^G12D^ /MYC^+^ cells. The data were analyzed using the log-rank test. χ2 = 3.900, *p* = 0.0483. **E.** Moderate hepatomegaly and splenomegaly in mice transplanted with both WT and KO NRAS^G12D^ /MYC^+^ cells. Grey boxes indicate the physiological weight range. **F.** Mice transplanted with either WT or KO NRAS^G12D^ /MYC^+^ cells develop a moderate anemia (RBC, red blood cells). White blood cells (WBC) and platelets (PLT) count in both cohorts shows a large variability. Grey boxes indicate the physiological range. **G.** Moribund animals transplanted with either WT or KO cells show expansion of NRAS^G12D^ /MYC^+^ cells in blood, BM and spleen, whereas only mice transplanted with KO NRAS/MYC^+^ cells display infiltration of lymph nodes (LN). **H.** Mice transplanted with WT NRAS^G12D^ /MYC^+^ cells develop a broad range of diseases. In mice receiving MEK1 KO cells, disease spectrum shifts towards myeloid malignancies. The asterisk in D and J indicates (*) one mouse with T-ALL, (**) the one mouse with edema, without any other symptoms of the disease. **I.** Immunoblot analysis of leukemic cell lines derived from hematological cancers. Tubulin A is shown as a loading control. tERK, total ERK; pERK, phosphorylated ERK. The Western blot is representative of 2 independent experiments.

To test the impact of MEK1 ablation in leukemias involving activation of both NRAS^G12D^ and MYC, we transplanted lethally irradiated mice with *Nras^G12D^*/*Myc* transduced cells (1% in WT protective BM; Figure 7A, 7C). Immunoblot analysis showed comparable expression of both NRAS and CMYC in GFP^+^/mCherry^+^ WT and KO cells, and increased ERK phosphorylation, slightly higher in the KO (Figure 7B). The recipients of MEK1 WT NRAS^G12D^/MYC^+^ BM cells developed a rapid disease (median survival time 24 days; Figure 7D) characterized by moderate hepatomegaly and splenomegaly (Figure 7E), moderate anemia, (Figure 7F), and expansion of NRAS^G12D^/MYC^+^ cells in blood (up to 45%), BM (up to 50%) and spleen (up to 60%; Figure 7G). Similar to the MYC-driven model, FACS analysis of WT NRAS^G12D^/MYC^+^ cells revealed a broad disease spectrum (MPD-like, mixed myeloid and B-cell malignancies, as well as T-ALL in one mouse; Figure 7H and Supplementary Figure 2). On the other hand, mice transplanted with MEK1 KO NRAS^G12D^/MYC^+^ cells exhibited significantly enhanced survival (mean survival time 33 day; *p* value = 0.0483 comparing mice receiving MEK1 WT and KO cells; Figure 7D). Moreover, recipients of MEK1 KO NRAS^G12D^/MYC^+^ cells showed exclusively myeloid disease (Figure 7H and Supplementary Figure 2). Additionally, in the MEK1 KO NRAS^G12D^/MYC cohort we observed enlarged lymph nodes containing Mac1^+^ Gr-1^+^ NRAS^G12D^/MYC^+^ cells (Supplementary Figure 2), reminiscent of myeloid sarcomas. Immunoblot analysis of leukemic cell lines derived from diseased mice showed variable, but comparable NRAS and MYC expression in lines derived from WT and KO animals (Figure 7I). The expression of both NRAS and CMYC was significantly higher in cells derived from T-ALL diseased WT mice, consistent with previous reports that T cells require stronger NRAS oncogenic signaling then myeloid cells [42]. ERK phosphorylation was slightly elevated in the KO leukemic cell lines, but the significance of this increase is limited by the different spectrum of transformed cells which could be obtained from recipients of WT and KO cells (Figure 7I). Taken together, MEK1 ablation prevented the development of NRAS^G12D^-driven disease, prolonged survival in NRAS^G12D^/MYC-driven leukemia model and shifted the spectrum of the latter disease towards myeloid malignancies.

The level of expression of oncogenic NRAS is a rate-limiting factor in leukemia development. In retroviral mosaic models such as the one used here, strong NRAS^G12D^ overexpression results in CMML-like MPD and AML with 3 months latency [43, 44]. In contrast, Mx-Cre-inducible expression of LSL-NRAS^G12D^ allele driven by its endogenous promoter led to late onset myeloproliferative diseases [45, 46], but did not develop acute leukemia. Similarly, mice homozygous for a hypomorphic NRAS^G12D^ allele do not develop tumors, while NRAS^G12D/G12D^ mice develop acute MPD within 3 months [42]. In further support of this, acquired uniparental disomy affecting the NRAS locus has been reported both in mouse models [46] and in patients [47], and correlates with the progression of the disease. While WT RAS has been previously reported to suppress the development of lung tumors [48] and thymic lymphomas [49], in leukemias WT NRAS lacks tumor suppressor activity, and acquired UPD serves the purpose of reaching the level of NRAS oncogenic pressure necessary for the disease progression [50].

Collectively, our data show that MEK1 is dispensable for the proliferation of human leukemia cell lines in culture or MYC-driven leukemogenesis; in contrast, MEK1 is required for the establishment of retroviral models of leukemia driven by NRAS alone or in combination with MYC. Essentially two mechanisms can be envisioned to rationalize the requirement for MEK in NRAS^G12D^-driven leukemogenesis. The first possibility is reduced signal flux through the MEK/ERK pathway. In other words, MEK1 might be required to obtain the full-fledged ERK activation necessary to fuel NRAS-driven leukemia. In line with this hypothesis, Aoidi and colleagues have recently shown that a certain level of MEK, irrespectively of the isoform, is necessary for proper activation of ERK signaling during mouse embryonic development [51]. In an analogous manner, MEK1 ablation might reduce the pool of MEK available for ERK activation in NRAS-driven leukemias and thus adversely affect the proliferative and/or survival capacity of the leukemic cells. The importance of full-fledged pathway activation has been recently underscored by the finding that myeloid leukemogenesis driven by endogenous RAS requires high levels of signaling that can be achieved by additional mutations disabling RAS negative feedback [52]. These observations are in line with our data showing complete protection from NRAS-driven T-ALL by MEK1 ablation, and with the finding that MEK inhibitors are able to suppress the growth of NRAS ^G12D^/^G12D^ T-ALL cells [42].

It is also possible, however, that the reduced leukemogenic activity of NRAS^G12D^ and NRAS^G12D^/MYC expressing MEK1 KO cells might be due to too high rather than too low signal levels. In non-transformed cells, MEK1 ablation increases both ERK and AKT signaling by disabling negative feedback steps in the pathways [14, 15]. Increased pathway activation correlates with the myeloproliferative disorder observed in the MEK1 KO mice, suggesting that MEK1 suppresses proliferation in this context. However, high levels of ERK signaling induced by oncogenic RAS have long been known to induce senescence and thus prevent transformation in culture and tumorigenesis *in vivo* until this failsafe mechanism is disabled [53-57]. Indeed, MEK inhibition or ERK2 silencing can bypass RAS-induced senescence in human fibroblasts and human mammary epithelial cells [58]. PI3K/AKT signaling can also induce senescence [59-61], albeit to a lower degree than RAS [62], and increased AKT activation as a consequence of PTEN heterozygosity or loss can induce tumor suppression in leukemia [63, 64]. It is thus possible that MEK1 ablation, by boosting two RAS downstream pathways connected to senescence, would raise the barrier to NRAS-driven leukemogenesis. In line with this, co-expression of MYC, which has been shown to suppress RAS- and RAF-induced senescence in tumorigenesis [65, 66], renders MEK1 KO BM cells permissive for leukemogenesis (Figure 6). While this hypothesis is attractive, it has to be noted that the pathways activated in MEK1 KO cells can cooperate to bypass senescence in prostate cancer cells [67] and in KRAS^G12D^-driven pancreatic tumorigenesis [62]. Finally, concurrent activation of RAF and AKT protects breast cancer cells from chemotherapy-induced senescence [68]. These observations would predict that deregulated signaling in MEK1 KO cells would counteract senescence, promoting tumorigenesis. Collectively, these reports highlight the complexity of the relationship between pathway crosstalk, signal output levels, and cell type-specific effects, illustrating the challenges the community is facing when dealing with signaling molecules as therapeutic targets [8].

In conclusion, our result show that loss of MEK1, while tumor-promoting in some contexts, can attenuate tumorigenesis in other contexts such as leukemias involving oncogenic NRAS activation. Thus, MEK1 does not act as a general tumor suppressor in leukemogenesis. Rather, its effects strongly depend on the genetic context (RAS versus MYC-driven leukemia) and on the cell type involved. Given the availability of advanced inhibitors and their efficacy in RAS-driven hematopoetic malignancies in both clinical and preclinical settings [12, 13], our data confirm that MEK should be further considered as a therapeutic target, in particular in combinatorial regimens with PI3K inhibitors. Previous attempts to treat patients with various advanced cancers with MEK inhibitors have been hampered by grade III-IV adverse effects of these drug, both as single agents [69] and in combination with PI3K inhibitors [70]. However, the latest phase II clinical trial with the new generation MEK inhibitor selumetinib in patients with advanced myelogenous leukemia has shown that the drug is reasonably well tolerated (adverse effects I-II grade), although it had only modest efficiency as a single agent [71]. The hope is that current efforts aimed at reducing the toxicity of MEK and PI3K or AKT inhibitors will render combination therapies feasible in the future.

## MATERIALS AND METHODS

### Ethics statement

The investigation has been conducted in accordance with the ethical standards and according to the Declaration of Helsinki and according to national and international guidelines. Animal experiments were authorized by the Austrian Ministry of Science and Communications, following the approval by the Ethical Committee for Animal Experimentation (permit number: BMWF-66.006/0002-II/3b/2014)

### Mice

*Mek1^f/f^* and *Mek1^f/f^; Sox2Cre* mice have been described previously [14]. Mice were backcrossed into the pure C57BL/6 background for 10 generations to obtain pure C57BL/6 *Mek1^f/f^; Sox2Cre* animals. *Mek1^f/f^;Vav1-iCre* mice were obtained by mating *Mek1^f/f^* C57BL/6-Ly5.2 mice with *Vav1-iCre* transgenic animals [72] provided by Meinrad Busslinger. Syngeneic C57BL/6-Ly5.1 recipient (maintained in the IMBA/IMP animal house) were used for *in vivo* leukemia studies.

### Plasmids constructs and cloning

Mouse *Myc* and *Nras^G12D^* cDNA were cloned into MSCV-based retroviral vectors [73, 74] co-expressing GFP (MSCV-IRES-GFP) or mCherry (MSCV-IRES-mCherry). The following vectors were generated: MSCV-MYC-IRES-GFP, MSCV-IRES-mCherry, MSCV-NRAS^G12D^-IRES-GFP. The helper packaging plasmid CMV-gag/pol was described previously [75].

20bp shRNAs against human MEK1 (*MAP2K1* mRNA (NM_002755.3); starting position: 1361, 1918, 857, 1916, 2100) were designed based on improved design rules [76] and cloned into microRNA stem (miR-E) in the pSIN-TRE3G-GFP-miR-E-PGK-Neo (RT3GEN) retroviral vector, as described previously [18]. A vector containing shRNA against renilla luciferase gene (Ren.713) served as control.

Guide RNA targeting human MEK1 exon 1 were designed using online CRISPR Design Tool software (http://crispr.mit.edu). The selected sequence (CGTTAACTGCAGAGCCGTCG) was designed as single strand DNA oligonucleotides containing overhangs suitable for cloning into BbsI restriction sites of pSpCas9(BB)-2A-GFP plasmid [77].

Retroviral constructs were propagated into Stbl3 *E.Coli* strain (NEB), cloning procedures and propagation of expression plasmids were performed in Top10 *E.Coli* (Invitrogene). All constructs were verified by sequencing and purified using QIAgen Plasmid Maxi Kit.

### Cell culture and proliferation assays

HL-60, K562 and THP-1 human leukemia cell lines were obtained from ATCC and cultured in RPMI1640 plus 10% FCS and penicillin/streptomycin (all from Sigma Aldrich). K562R and THP-1R cell lines, modified to express ecotropic receptor and rtTA3 as described previously[17], were grown under the same conditions as the parental lines with addition of puromycin (Sigma Aldrich) (1.5μg/ml or 0.75 μg/ml, respectively). To generate MEK1 knock-down (KD) cell lines, K562R and THP-1R cells were transduced with RT3GEN based retroviral pseudoparticles supplemented with 4 μg/ml polybrene and spinfected for 30 min at 450xg, 37°C. The infection was repeated after 5, 10 and 24 hr. Transduced cells were selected for two weeks in 1 mg/ml G418 (Invitrogene). Positively selected cells were treated with 1 μg/ml of doxycycline for 72hr to induce expression of the GFP-miRE cassette and thus MEK1 knock-down.

To generate MEK1 knock-out cells, HL-60 cells were transiently nucleofected with pSpCas9(BB)-2AGFP containing sgRNA against *MEK1* exon1 using Amaxa^®^ Cell Line Nucleofector^®^ Kit V (Lonza), according to the manufacturer's protocol. Viable, GFP^+^ cells were single-cell sorted into 96-well plate and cultured for three weeks. The plates were screened by light microscopy for the presence of growing clones, which were further expanded. MEK1 KO clones were identified by immunoblotting and the knock-out was further confirmed by sequencing of the Cas9-targeted site in the *MEK1* gene PCR amplified using primers for hMEK1exon1 (forward: GGTTGGTTGAGAGAGAGAGAGG; reverse: GCTGGTCTCAAAAGCACAGATG).

To monitor proliferation, THP-1R MEK1 KD and Ren ctr cell lines were seeded in 6 wells of a 96-well plate at the concentration of 2x10^4^ cells/well in 200 μl of medium. Half of the wells were treated with 1 ug/ml of doxycycline (triplicates for each condition). After 72hr incubation, the cells were resuspended thoroughly and 50 μl of the cell suspension were transferred to a new plate, fed with additional 50 ul medium and cultured for an additional 4 days. The cells were allowed to metabolize MTT (0.6mg/ml; Sigma Aldrich) for 2hr at 37°C, 5% CO_2_. After incubation the formazan crystals were solubilized with 110 μl of acidified isopropanol (0.04M HCl in absolute isopropanol) and the absorbance was measured at 595 nm with a Victor3V plate reader (Perkin Elmer).

HL-60 ctr and MEK1 KO clones were seeded at the concentration of 1x10^5^ cells/ml, cultured for 24, 48 or 72 hr, at 37°C, 5% CO_2_ and counted. The experiment was repeated 4 times.

For competition assays, K562R cells, K562R MEK KD (MEK1.1361, MEK1.1918, MEK1.857, MEK1.1916, MEK1.2100) and K562 Ren.713 ctr cells were pretreated with 1 μg/ml of doxycycline for 72hr. K562R MEK1 KD or Ren ctr cells (all expressing GFP-miR-E after doxycycline induction) were mixed 1:1 with K562R cells (no GFP-miR-E cassette). The cell mixture was seeded at the final concentration of 1x10^5^ cells/ml in 2 ml of RPMI plus 0.5 or 10% FCS and penicillin/streptomycin. The cells were incubated at 37°C, 5% CO_2_ and passaged every 2-3 days for a period of 3 weeks. The percentage of GFP^+^ cells in the initial inoculum and after each passage was determined by flow cytometry (FACSCalibur, BD Biosystems).

### Generation of high-titer retroviral pseudoparticles

The Platinum-E packaging cell line (Cell BioLabs) was maintained in DMEM plus 10% FCS and penicillin/streptomycin (all from Sigma Aldrich).

Ecotropic retroviral pseudoparticles were produced in Platinum-E cells, essentially as previously described [78]. Briefly, 8-10hr before transfection, a 90% confluent ø10 cm plate of Platinum-E cells was split 1:2. Packaging cells were transfected using a standard calcium phosphate co-precipitation method. After 12hr, the transfection medium was exchanged. After 12hr incubation, the medium was replaced with 5 ml of medium optimal for transduced cells. Retrovirus-containing supernatants were harvested 36-48hr after the transfection, filtered through 0.45-μm syringe filter and used freshly to transduce target cells.

### Bone marrow cell transduction

Bone marrow (BM) cells were isolated from femurs, tibias and hip bones of 6-9 weeks old mice using a crushing method. Red blood cells (RBCs) were lysed for 2 min at RT in 2 ml of RBC lysis buffer. Bone marrow stem and progenitor cells enrichment (BM HSPCs; enrichment for cKit^+^) was performed using mouse CD117 Microbeads (Miltenyi biotec) according to the manufacturer's protocol. 1x10^6^ cKit^+^ BM cells were seeded in 6-well plate in 1.2 ml of StemPro-34 SFM medium (Gibco) supplemented with 1X StemPro-Nutrient Supplement, 2 mM Glutamax (Gibco), penicillin/streptomycin and a cytokine cocktail optimized for expansion of HSPCs: 2 ng/ml mrIL-3 (R&D Systems), 2 ng/ml hrIL-6 (eBioscence), 10 ng/ml mrSCF (R&D Systems), 2 ng/ml mrTPO (R&D Systems), 10 ng/ml mouse recombinant Flt-3 ligand (R&D Systems) [31]. After 18hr of pre-culture, BM HSPCs were transduced with 1 ml of MSCV-based retrovirus-containing supernatants supplemented with 4 μg/ml polybrene (Sigma Aldrich), 1 mM dNTPs (Thermo Scientific) and cytokines, and spinfected for 30 min at 450xg, at 37°C. The infection was repeated after 5, 10 and 24 hr. 24hr after the last infection, BM cells were harvested, washed twice in cold PBS and used immediately for further *in vivo* or *in vitro* experiments.

### Colony forming assay

Transduced BM cells from *Mek1^f/+^* (WT) or *Mek1^f/f^; Sox2Cre*(KO) were stained with Fixable Viability Dye eFluor 450 (eBioscience) and for each condition 6x10^3^ viable, GFP^+^ cells were sorted on FACSAria- IIIu LSR (BD Bioscience) directly into 3.3 ml of MethoCult M3434 semisolid media (StemCell Technologies). Cells were seeded in duplicates in 6-well plates (2x10^3^ cell/well in 1.1ml of medium) and cultured for 10 days at 37°C, 5% CO2. Following the incubation, the number of colonies was determined. The cells were harvested, counted and replated in fresh MethoCult M3434 (2x10^3^ cell/well in 1.1ml of medium). Counting and replating was repeated 3 times.

### *In vivo* leukemia studies

BM cells from 6-9 week old *Mek1^+^/^+^; Vav1-iCre ^+^*(WT) or *Mek1^f/f^; Vav1-iCre^+^* (KO) mice were cotransduced with the following retroviral pseudoparticles: a) MSCV-MYC-IRES-mCherry and MSCVGFP; b) MSCV-GFP-IRES-NRAS^G12D^ and MSCV-mCherry; c) MSCV-MYC-IRES-mCherry and MSCV-GFP-IRES- NRAS^G12D^. Viable, transduced cells were sorted on FACSAria-IIIu LSR and either used for immunoblot analysis or mixed with wild type (WT) protective BM. 8-12 week old female C57BL/6-Ly5.1 recipients were lethally irradiated with 2 doses of 5.5 Gy (first in the previous evening and secondly directly before the injections). Mice were injected intravenously via the tail vein with 1x10^6^ protective BM per mouse containing a percentage of transduced cells adjusted to result in disease kinetics enabling the detection of a phenotype. Mice were monitored daily for disease symptoms and moribund animals were sacrificed with CO_2_. Primary and secondary hematopoietic organs (BM, spleen, blood, thymus and lymph nodes) were harvested, the cells isolated in IMDM plus 2% FCS and penicillin/streptomycin, and further analyzed by FACS. The log-rank test was used to evaluate the significance of the difference in survival. A p value ≤ 0.05 is considered statistically significant.

### Blood analysis and flow cytometry

Peripheral blood counts were acquired using the V-Sight hematology analyzer (A. Menarini Diagnostic). RBCs were lysed for 5 min at RT in RBC lysis buffer. Freshly isolated BM, spleen, thymus, lymph node, and white blood cells were stained with Fixable Viability Dye eFluor 780 (eBioscience), blocked for 10 minutes with TruStain FcX anti-mouse CD16/32 (BioLegends) and stained for 30 minutes in brilliant stain buffer (BD Bioscience) with antibodies against Ly5.1-PerCP-Cy5.5 (eBioscence #45-0453), Ly5.2-V500 (BD Bioscence 562129), CD19-BV786 (BD Bioscience #563333), CD3e-APC (BD Bioscence #553066), Mac1-BV650 (BD Bioscience #563402), Gr-1-APC (eBioscience #17-5931) and Ter119-BV421 (BD Bioscence 563998). Cells were analyzed by FACSFortessa (BD BioScience) and FlowJo software.

### Whole cell lysates and immunoblotting

Leukemic cells lines were harvested by centrifugation at 300xg for 5 minutes, lysed in buffer containing 20 mM TrisHCl pH 7.4, 137 mM NaCl, 1 mM CaCl2, 1 mM MgCl2, 1% NP-40, 1 mM Na3VO4, 50 mM NaF, 2 mM PMSF and protease inhibitor cocktail (Roche). Transduced, sorted BM cell were lysed in 1X Laemmli buffer supplemented with 1 mM Na3VO4, 50 mM NaF, 2 mM PMSF and protease inhibitor cocktail. The total protein concentration was measured using Pierce BCA Protein Assay Kit (Thermo Scientific). 5-20 μg of protein were analyzed on 10% SDS-PAGE gels. The proteins were transferred to ImmobilonP PVDF membranes (Milipore) activated in ethanol, by overnight wet blotting in Tris-Glycine. The membranes were blocked for 1hr in 5% milk in TBS-T, and incubated for 1hr at room temperature or overnight at 4°C with primary antibodies (MEK1 (Cell Signaling #2352), MEK2 (BD Transduction Laboratories #610236), GFP (Roche #11814460001), GAPDH (Milipore #abs16), TUBA (Sigma #T9026), tERK (Cell Signaling #9102), pERK(Cell Signaling #9101), CMYC (Abcam #ab32072) and NRAS (Santa Cruz #sc-31) and developed using SuperSignal West Pico/Femto Chemiluminescent Substrate (Thermo Scientific), Amersham Hyperfilm (Ge Healthcare) and CURIX 60 processor (Agfa) or ChemiDoc Touch Imaging System (Bio-Rad). Densitometric quantification of immunoblots was performed using ImageLab software.

## Supplemental Data


