# Supplementary material for: MEK1 is required for the development of NRAS-driven leukemia
Source: Oncotarget. 2016 Oct 10;7(49):80113–30. doi: 10.18632/oncotarget.12555 (PMC5348309; doi:10.18632/oncotarget.12555)
Supplement: Supplementary file 1 [file oncotarget-07-80113-s001.pdf]

MEK1 is required for the development of NRAS-driven leukemia

Supplemental Data

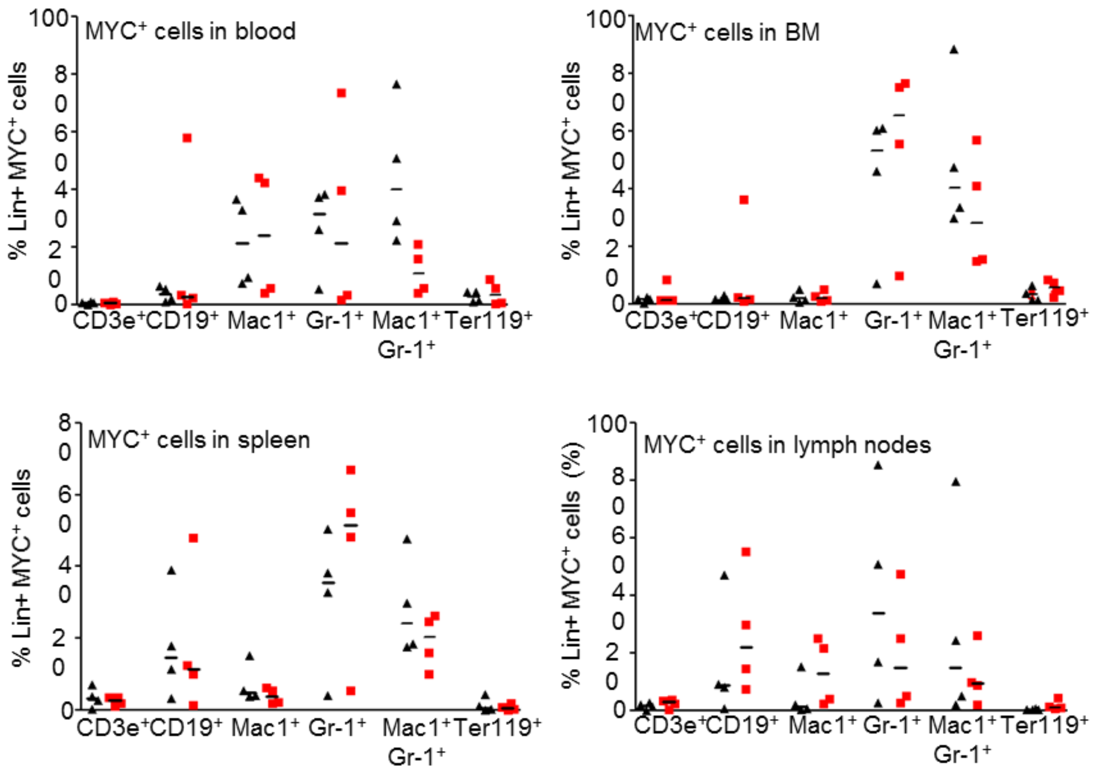

Supplementary Figure 1. Lineage distribution of MYC^{+} cells.

The graphs show the percentage of MYC^{+} cells positive for the indicated lineage marker in blood, BM, spleen and lymph nodes.

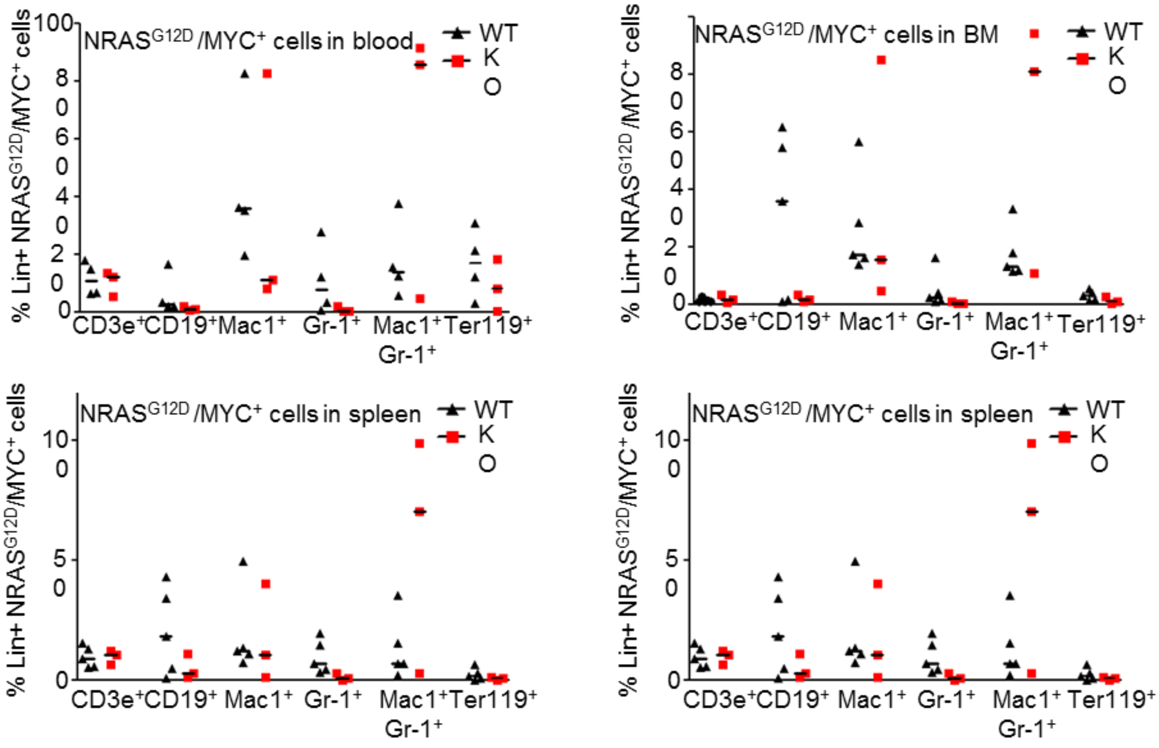

Supplementary Figure 2. Lineage distribution of NRAS^{G12D}/MYC^{+} cells.

Graphs show the percentage of NRAS^{G12D}/MYC^{+} cells positive for the indicated lineage marker in blood, BM, spleen and lymph nodes.
